# Canon Newbolt on Suffering and Good-Will

**Published:** 1892-04-23

**Authors:** 


### The Hospital

AN INSTITUTIONAL JOURNAL OF

Science, Medicine, Nursing, and Philanthropy.

*For Hospitals, Asylums, Infirmaries, Dispensaries, Medical Practitioners, Students, Nurses, and the Charitable Public.*

VOL. XII.—NO. 291.] [APRIL 23, 1892.

### CANON NEWBOLT ON SUFFERING AND GOOD-WILL.

THE Rev. Canon Newbolt, preaching at St. Paul's Cathedral on Palm Sunday, expressed the conviction that there is enough good-will among the prosperous in London and elsewhere to deal effectually with the suffering and distress which are so urgent all around us, if only right methods could be devised. This is an extraordinary proposition. Is it true? Is it really the want of intellectual capacity alone that prevents us putting an end to all preventable and curable suffering and distress? "There is one thing to remember," said the preacher, "that unrelieved distress and great social depression and difficulties in trade do not mean a selfish indifference to human misfortune on the part of those who stand by. There are thousands of people ready to help, longing to help, yearning to help, if only the problem could be solved how best to do it. Suffering and good-will, those are the two phenomena, alas! both apparent. The problem is how to bring them together without loss of self-respect on the one hand or mere perfunctory dole-giving on the other."

There are some who see the suffering of which Canon Newbolt speaks quite as clearly as he does, but they are by no means so confident about the thousands upon thousands who are "longing and yearning" to help. There can be no doubt about the many thousands who are "longing and yearning" not to help, and who find no difficulty whatever in putting the yearning into effectual practice. The English, as we are so constantly told, are above all things a practical people; and the sight of thousands of them looking on at distress and wringing their hands in helpless despair because they do not know how to set themselves to the work of relief is a picture which may be very clear to Canon Newbolt's imagination, but which has exceedingly little resemblance to the actual facts of the case.

The thing which strikes many of us in connection with the "good-will" of a small portion of the more prosperous of the English people is, not their despairing helplessness in the presence of suffering and distress, but their resolute and business-like capacity in dealing with the distress and vanquishing it. Canon Newbolt was probably trying to administer a little consolation to those members of the working classes who are out of work at the present time, and who suffer in consequence. But the out-of-works are not the only members, even of their own class, who know what suffering and distress are. The workman who is disabled by sickness or accident, and is compelled to seek the shelter of a hospital is in a much worse plight than the man who is merely out of work. The sick or the injured man is not able to work, though his wife and children, as well as himself, require to be fed and housed all the same. A small portion of the prosperous part of the community, in taking charge of the sick and injured, renders a service to the working-class generally which is of a magnitude that has never yet been even approximately realised. When a workman breaks his leg, or is attacked by prostrating fever, as is the case with hundreds of the class every week, there is no piteous wringing of hands, or despairing cries, asking what is to be done with the poor fellow? The business-like "goodwill" of the practical few who mean what they say has planted homes for the sick in almost every part of our thickly-populated towns, and maintains those homes with wide-open doors for every case of helpless suffering which may fall down on the highway of life. If the working classes, in addition to the distresses which come in the train of loss of employment, were compelled by the non-existence of hospitals to maintain themselves in sickness, and to endure the agonies of the poverty thus brought upon them, as well as the sufferings incidental to their diseases, the lot of many of them would be well nigh unendurable. But all this they are delivered from, and delivered from by the sympathy and help of a limited number of practical philanthropists, who have put their shoulders to the wheel in such a sturdy and resolute way that they have placed at the disposal of the sick workman the very highest resources which modern science can lay her hands upon. There is surely no incapacity here.

It must not, then, be said to the workman that the prosperous would help him if they only knew how. Some of them do know how; they do help him; they will help him more; only if the help is to effectually save him from every form of preventable distress, he must make some effort to help himself a very little bit. The workman should ask himself how it is that so many members of the middle-class are in the position of giving help to him, rather than in that of asking it for themselves? A large proportion of the middle class would not have been able to give anything in charity at all if they had managed the business of their lives as working men manage their business. The workman marries early; the middle-class man marries late. The workman marries without thought and without provision; the middle-class man provides himself a home, with some resources, and some prospect of continuance, before he takes to himself a wife. The workman does not believe in self-denial or in patience; he

ARMY MEDICAL LIBRARY

hurries to indulge his appetites and his passions; the middle-class man inflicts abstinence upon himself; he resists his appetites; he keeps his passions in subjection; he endures storms and buffetings for a few years in early manhood, in order that he may have calm seas in middle age, and a quiet haven at the end of life.

When the workman is actually out of work, without money in his pocket, and has a wife and children at home starving, then is the time for sympathy and not for reproaches. But still it must be borne in mind that in this world every one of us is under the necessity of fighting for his own hand. If the workman insists on depending upon others rather than upon himself, and if he will, at all costs, indulge his passions and his appetites as they spring up within him, without any attempt at self-restraint, his helplessness will increase upon him at such a rate that the other classes, however willing they may be, will find themselves absolutely unable to cope with the gigantic task which is by consequence placed before them. With Canon Newbolt we agree, that, at any rate, some of the prosperous are willing to help the unprosperous; but, unlike Canon Newbolt, we maintain that they are capable and effectual in their help, though they cannot do impossibilities.

It would be well at this time to call to mind what has been practically effected in London generally for the sick portion of the working classes, and in one part of London, at any rate, for the distressed and the out-of-work. We are informed that at Paddington out-of-work people are practically unknown, and that unrelieved hunger and poverty do not exist. In that parish a very simple plan has been found efficacious. All classes and denominations of the prosperous have unanimously conjoined their forces; a central committee has been formed; certain voluntary workers work loyally under the direction of the central committee. Every small house in the parish is systematically and frequently visited; those persons who are out of work are provided with temporary or permanent employment; those who have no money are supplied with money, food, and other necessaries until they are able to provide for themselves. All this is done in Paddington, and not merely talked about. The things that most surprise the better classes in that part of London are, that though there is a large working class population, there are very few out-of-works, and an exceedingly small number who are found to be in genuine distress. The money, however, even in this case, is supplied by a few; the "yearning" many preferring to pay in expressions of "the warmest sympathy."

Those who make the loudest noise are usually the first to be attended to. But the philanthropists of England, who are doing a noble work for their country, cannot afford to be run away with by panic. They must do the work which they have already taken in hand, and which they know to be absolutely necessary and absolutely good. However loud an outcry may be made by those who say they are out of work, or by any other class, we must not put down the philanthropic business which we have already taken up, and which experience has taught us how to work so admirably, merely for the sake of running after work of which we know little or nothing, and which may be neither half so necessary nor half so important as that we already have in hand. As we have said, in providing for the sick and injured we have relieved the working classes of an enormous burden; and the out-of-works themselves have their share in the benefit of the relief. Of course, we are among the first to advocate the claims of the genuine out-of-works; and it is with the view of furnishing guidance to those who may be striving to help them that we cite the case of Paddington, and record what has been successfully done there. But we repeat that the practical Briton has been, and is, as practical in his philanthropy as in other things; and in incontestible proof of this we adduce the existence and daily work of the London and country hospitals.

